# Medicaid Managed Care and Pediatric Dental Emergency Department Visits

**DOI:** 10.1001/jamahealthforum.2024.1472

**Published:** 2024-06-14

**Authors:** Lawrence Baker, Elizabeth L. Munnich, Ashley M. Kranz

**Affiliations:** 1 RAND Corporation, Boston, Massachusetts; 2 Pardee RAND Graduate School, Santa Monica, California; 3 Department of Economics, University of Louisville College of Business, Louisville, Kentucky; 4 RAND Corporation, Arlington, Virginia

## Abstract

**Importance:**

Millions of economically disadvantaged children depend on Medicaid for dental care, with states differing in whether they deliver these benefits using fee-for-service or capitated managed care payment models. However, there is limited research examining the association between managed care and the accessibility of dental services.

**Objective:**

To estimate the association between the adoption of managed care for dental services in Florida’s Medicaid program and nontraumatic dental emergency department visits and associated charges.

**Design, Setting, and Participants:**

This cohort study used an event-study difference-in-differences design, leveraging Florida Medicaid’s staggered adoption of managed care to examine its association with pediatric nontraumatic dental emergency department visits and associated charges. This study included all Florida emergency department visits from 2010 to 2014 in which the patient was 17 years or younger, the patient was a Florida resident, Medicaid paid for the visit, and a primary or secondary *International Classification of Diseases, Ninth Revision, *code was used to classify a nontraumatic dental condition. Analyses were conducted between May 2023 and April 2024.

**Exposure:**

The county of residence transitioning Medicaid dental services from fee-for-service to a fully capitated managed care program managed by a dental plan.

**Main Outcomes and Measures:**

The rate of nontraumatic dental emergency department visits per 100 000 pediatric Medicaid enrollees and the associated mean charges per visit. Nontraumatic dental emergency department visits are a well-documented proxy for access to dental care. Data on emergency department visit counts came from the Florida Agency for Health Care Administration. Medicaid population denominators were derived from the American Community Survey’s 5-year estimates.

**Results:**

Among the 34 414 pediatric nontraumatic dental emergency department visits that met inclusion criteria across Florida’s 67 counties, the mean (SD) age of patients was 8.11 (5.28) years, and 50.8% of patients were male. Of these, 10 087 visits occurred in control counties and 24 327 in treatment counties. Control counties generally had lower rates of NTDC ED visits per 100 000 enrollees compared with treatment counties (123.5 vs 132.7). Over the first 2.5 years of implementation, the adoption of managed care was associated with an 11.3% (95% CI, 4.0%-18.4%; *P* = .002) increase in nontraumatic dental emergency department visits compared with pre-implementation levels. There was no evidence that the average charge per visit changed.

**Conclusions and Relevance:**

In this cohort study, Florida Medicaid’s adoption of managed care for pediatric dental services was associated with increased emergency department visits for children, which could be associated with decreased access to dental care.

## Key Points

**Question:**

What is the association between the adoption of Medicaid managed care for dental services and pediatric dental emergency department visits in Florida?

**Findings:**

In this cohort study of 34 414 nontraumatic dental emergency department visits among children enrolled in Florida Medicaid from 2010 to 2014, the introduction of a managed care plan for Medicaid dental services in Florida was associated with an 11.3% increase in the number of pediatric emergency department visits for nontraumatic dental conditions.

**Meaning:**

Medicaid managed care plans may reduce access to dentists relative to fee-for-service arrangements.

## Teaser

This cohort study estimates the association between the adoption of managed care for dental services in Florida’s Medicaid program and nontraumatic dental emergency department visits and associated charges.

## Introduction

Children enrolled in Medicaid are entitled to comprehensive dental care through the federal Early and Periodic Screening, Diagnostic, and Treatment benefit. Despite this benefit, many Medicaid-enrolled children do not routinely access dental care, with only 45% of eligible children receiving any dental services in 2021.^1^ Low rates of utilization stem from multiple sources, including low dentist participation, concerns about reimbursement rates^2^ and administrative complexity,^3,4^ and long wait times.^5^ Access is particularly limited in rural communities, where there are fewer dentists and practices tend to be smaller and therefore less able to navigate the administrative burden associated with accepting Medicaid.^6,7,8^

Some children with nonemergency dental needs seek care at emergency departments (EDs).^9^ National nontraumatic dental condition (NTDC) ED visit rates average 68.3 per 100 000 Medicaid-enrolled children younger than 15 years.^10^ NTDC ED visit rates are elevated among Medicaid enrollees, those who are uninsured, and young adults.^10,11^ EDs typically cannot treat the underlying cause of the NTDC and instead alleviate symptomatic pain, so ED use is considered expensive for the health care system and ineffective for the patient.^11,12^

In an attempt to control costs and improve access to dental services,^13^ many states have transitioned Medicaid dental benefits to fully capitated payment models (hereafter referred to as managed care). In these programs, private medical or dental plans take on the financial risk of providing dental services in return for risk-adjusted capitated payments. Some states operate programs in which case management or administrative services are capitated but care is reimbursed under a fee-for-service (FFS) model; these programs are more closely aligned with FFS. The transition to managed care is ongoing—as of 2018, 18 states (listed in the eAppendix in Supplement 1) still administered pediatric Medicaid dental benefits through a FFS payment model,^14^ and since that time, several states have initiated or considered new managed care programs.^15,16,17,18^

The theoretical effect of managed care programs is ambiguous. Proponents argue that managed care programs improve coordination between medical and dental services and incentivize delivery of preventive care with the goal of avoiding expensive restorative treatment.^19^ Critics argue that they increase administrative burdens and create incentives for plans to limit access.^20^ Empirically, medical Medicaid managed care programs have an inconsistent record of improving care or reducing costs,^21,22,23^ but there is limited evidence on the effect of dental Medicaid managed care programs. One study examined whether transitioning Medicaid pediatric dental benefits from FFS to managed care changed service utilization in Indiana, Missouri, and Nebraska compared with states that retained FFS.^14^ Overall, switching to managed care was associated with a 2.6% drop in the share of Medicaid enrollees with a dental claim. However, results varied considerably by state. Dental claim rates dropped sharply in the first 2 quarters in Indiana before recovering. Reductions were more modest in Nebraska and nonsignificant in Missouri.

The present study builds on prior work by analyzing how NTDC ED visits changed during the introduction of managed care for Florida Medicaid’s pediatric dental services from 2010 to 2014, a program known as prepaid dental health plans (PDHPs). Under FFS, all services were billed by dentists to the state according to a fee schedule. Under PDHPs, 2 dental plans, MCNA Dental and Delta Dental, received a capitated rate from the state and were each responsible for forming a network of dentists, providing care, and negotiating fee schedules. Medicaid enrollees were allocated to one of these plans, which subsequently assigned them to an in-network dentist. Dental plans may have achieved cost containment through negotiating lower rates with dentists, limiting networks to low-cost dentists, reducing utilization, expanding preventive services that reduce need for restorative services, or identifying administrative efficiencies.

For outcomes, we focused on NTDC ED visits because they directly measure the use of a costly and usually ineffective form of care and are a proxy for dental care accessibility.^11^ When seeking treatment for painful and intractable dental conditions, families must choose whether to visit their dentist or the ED. As accessing a dentist becomes more difficult, more families choose the ED, increasing the rate of NTDC ED visits. We used an event-study difference-in-differences (DiD) design to estimate changes in the NTDC ED visit rate and the associated charges following the adoption of managed care. We hypothesized that the introduction of managed care disrupted access to dental services because patients could only receive care from in-network dentists rather than any dentist who accepted Medicaid, increasing NTDC ED visits.

## Methods

### Data, Sample, and Variables

We constructed the analytic dataset using data on ED visits and the Medicaid population. Quarterly ED visit and charge data were obtained from the Florida Agency for Health Care Administration (AHCA). These data cover all ED visits in Florida. Records include patient demographics, self-classified race and ethnicity, county of residence, payer information, diagnoses (≤10 *International Classification of Diseases, Ninth Revision,* codes), and total charges.^24^ We obtained and used records from the first quarter of 2010 through the second quarter of 2014, after which Florida Medicaid made additional policy changes to how it covered dental care. Data on the number of Medicaid enrollees 17 years or younger in Florida were extracted from the American Community Survey (ACS) 5-year data^25^ using the tidycensus R package.^26^ For additional details on the preparation of population data, see eTable 1 and eFigure 1 in Supplement 1.

We used NTDC ED visits as a proxy measure for dental care accessibility. NTDCs encompass conditions such as dental caries, abscesses, inflammation, and related pain. Each NTDC ED visit suggests a breakdown in dental care access because these conditions are both preventable and best treated in a dental office. NTDCs have widely been used to study dental health policy changes, including Medicaid expansion^27,28,29,30^ and the elimination of Medicaid dental benefits.^31,32,33^

To measure counts of pediatric NTDC ED visits covered by Medicaid, we subset ED visit data using the following inclusion criteria: (1) the patient was 17 years or younger; (2) the patient was a Florida resident (2.3% of pediatric NTDC visits without a resident Florida county code were excluded); (3) Medicaid paid for the visit; and (4) a primary or secondary *International Classification of Diseases, Ninth Revision,* code was classified as an NTDC per the Association of State and Territorial Dental Directors’ guidelines.^34^ We identified when counties adopted managed care using the Florida Medicaid monthly enrollment report from the AHCA.^35^ Where multiple monthly files were available for the same quarter, the mean was used.

The primary outcome was the number of NTDC ED visits per 100 000 Medicaid enrollees 17 years or younger. The secondary outcome was the mean charge per NTDC ED visit among this population.

The RAND Human Subjects Review Committee deemed this analysis exempt from additional review because it was secondary research for which patient informed consent was not required. We followed the Strengthening the Reporting of Observational Studies in Epidemiology (STROBE) reporting guidelines for cross-sectional studies.

### Statistical Analysis

This analysis focused on the shift from FFS to managed care for Florida Medicaid’s pediatric dental services. Most of Florida’s counties (61 of 67) first adopted managed care for pediatric dental services between the first quarter of 2012 and the first quarter of 2013. We included these counties in the treatment group. The remaining 6 counties implemented managed care for dental services much earlier, between 2004 and 2007, as part of 2 separate pilot programs: one for PDHPs and one for a comprehensive managed care system that included medical and dental benefits.^36,37^ We used these pilot counties as controls because their payment structures did not change during the 2010 to 2014 analysis period. We assumed that there were no remaining treatment dynamics in control counties because they adopted managed care more than 5 years before the treatment counties. Table 1 summarizes Medicaid in each county that first adopted managed care for pediatric dental care among Medicaid enrollees. eFigure 2 in Supplement 1 shows enrollment in managed care dental plans in each treatment county.

**Table 1.** Timing of the Introduction of Managed Care for Pediatric Dental Services in Florida Counties

Counties	Adopted managed care for dental services	Study group
Miami-Dade	2004	Control
Broward, Duval	2006	Control
Nassau, Baker, Clay	2007	Control
Indian River, Martin, Okeechobee, Palm Beach, St Lucie	2012, First quarter	Treatment
Brevard, Hardee, Highlands, Hillsborough, Manatee, Orange, Osceola, Pasco, Pinellas, Polk, Seminole	2012, Fourth quarter	Treatment
Remaining 45 counties	2013, First quarter	Treatment

We estimated a DiD event study using a two-way fixed effects (TWFE) model with county-quarter observations. We weight the regression by the number of pediatric Medicaid enrollees in each county-quarter. This regression yielded average treatment effect on the treated (ATT) estimates for each treatment group in each quarter. We calculated the overall ATT as the weighted average of all posttreatment groups. We constructed standard errors and the corresponding pointwise confidence intervals using 100 000 iterations of county-clustered multiplier bootstrapping. We did not add additional controls beyond the treatment and fixed effects required for TWFE because including covariates that are not orthogonal to treatment can induce bias in event-study designs.^38^

Recent DiD literature highlights that TWFE can yield biased estimates when applied to a staggered policy intervention if there are time-varying or heterogeneous effects.^39,40,41^ We used a method proposed by Callaway and Sant’Anna^41^ to correct for these biases, implemented with the did R package, version 2.1.2.^42^

As robustness checks on the primary outcome results, we estimated several alternative model specifications, which are described in detail in the eAppendix in Supplement 1. Specifically, we aggregated the county-quarter observations into a simplified 2 × 2 DiD model with 2 time periods (preintervention and postintervention) and 2 groups (treatment and control). We also present results using NTDC ED visit counts, rather than NTDC ED visit rates, to verify that changes in rates are primarily associated with changes in the number of visits (the numerator) rather than changes in the Medicaid population (the denominator). Because Florida counties vary greatly in size, we compared percentage changes in counts, rather than absolute changes in counts.^43^ Finally, because there is no consensus on the optimal approach for adjusting TWFE estimates in the presence of staggered rollouts,^44^ we present ATTs from 3 alternative methods (Wooldridge,^39^ Sun and Abraham,^45^ and Gardner^46^).

All analyses were conducted with R, version 4.2.3 (R Foundation for Statistical Computing). A 2-sided *P* < .05 was considered statistically significant.

## Results

A total of 34 414 NTDC ED visits were analyzed after meeting inclusion criteria. Of these, 10 087 visits occurred in control counties and 24 327 in treatment counties. A comparison of these counties is summarized in Table 2, focusing on pretreatment periods from the first quarter of 2010 to the fourth quarter of 2011.

**Table 2.** Summary Statistics for Control and Treatment Counties in Pretreatment Quarters^a^

Characteristic	Counties, %	
	Control^b^	Treatment
No. of observed NTDC ED visits^c^	24 327	10 087
NTDC dependent variables		
No. of quarterly NTDC ED visits per 100 000 pediatric Medicaid enrollees	123.5	132.7
Charge per pediatric NTDC visit, mean (SD), $	823 (972)	841 (1210)
Mean county population of Medicaid enrollees <18 y	72 187	15 286
Patient characteristics across NTDC ED visits		
Age, mean (SD), y	7.39 (5.31)	8.59 (5.39)
Sex		
Female	51.4	48.7
Male	48.6	51.3
Race and ethnicity^d^		
Hispanic	33.2	21.5
Non-Hispanic Asian	0.3	0.6
Non-Hispanic Black	48.1	34.0
Non-Hispanic White	15.3	41.1
Non-Hispanic other	2.8	2.1
Unknown	0.3	0.6

Abbreviations: ED, emergency department; NTDC, nontraumatic dental condition.

^a^ Includes data for 6 control counties and 61 treatment counties in Florida. Except for the number of observed NTDC ED visits, the variables shown are for the pretreatment periods from the first quarter of 2010 until the fourth quarter of 2011.

^b^ Control counties are those that received the prepaid dental health plan pilot or the statewide Medicaid managed care pilot.

^c^ From the first quarter of 2010 until the second quarter of 2014.

^d^ Race and ethnicity data, which were self-reported or reported by a parent or guardian, were analyzed to determine whether the treatment and control counties had similar demographic compositions. The Non-Hispanic other category includes individuals who reported as Native Hawaiian or Other Pacific Islander or other. They were grouped together owing to small sample sizes.

Control counties generally had lower rates of NTDC ED visits per 100 000 enrollees compared with treatment counties (123.5 vs 132.7). This difference was primarily associated with the notably lower rate in Miami-Dade County. The average charges per NTDC ED visit were similar across both groups, with treatment counties averaging $841 and control counties $823. Control counties, compared with treatment counties, generally had larger mean populations (72 187 vs 15 286) and a smaller proportion of ED visits for non-Hispanic White residents (15.3% vs 41.1%).

There was a large and statistically significant increase in NTDC ED visits among pediatric Medicaid enrollees following the implementation of managed care. Specifically, there was an increase of 15.0 visits (95% CI, 5.4-24.5 visits; *P* = .002) per 100 000 enrollees per quarter after managed care was introduced in treatment counties, which translates to an additional 59.8 visits (95% CI, 21.4-98.2 visits) per 100 000 enrollees annually. This represents an 11.3% (95% CI, 4.0%-18.4%) increase in visits compared with the pretreatment average in treated counties. These results are similar when only visits with a principal NTDC diagnosis are considered, as summarized in eTable 3 in Supplement 1. Additionally, there was no statistically significant association between managed care and the mean charge to Medicaid for NTDC ED visits, with an ATT of −$49.09 (95% CI, −$143.15 to $44.97; *P* = .31).

The quarterly ATTs shown in the Figure and eTable 4 in Supplement 1 consistently increase over time after managed care was implemented, though most quarterly estimates are not individually statistically significant due to limited sample sizes. In addition, pretreatment ATTs are nonsignificant and small relative to posttreatment ATTs, supporting the DiD parallel trends assumption.

**Figure.**
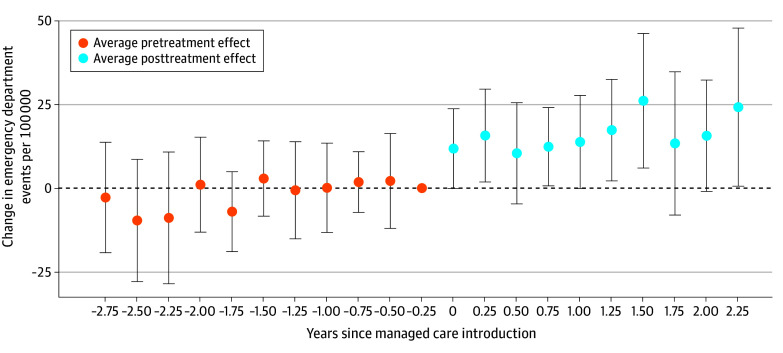
Quarterly Event Estimates of the Association Between Managed Care and Pediatric Nontraumatic Dental Condition Emergency Department Visits Data include 67 counties in Florida, 1206 county-quarters, and 34 414 nontraumatic dental condition emergency department visits. Pretreatment and posttreatment average treatment effects are constructed using long differences. Dots indicate difference-in-differences point estimates, and error bars indicate 95% CIs.

To evaluate the robustness of these findings, several alternative model specifications were estimated, with overall ATTs summarized in Table 3. The ATT for the simplified 2 × 2 DiD specification was statistically significant and similar in magnitude to the main analysis. This suggests that the primary results are representative of treated counties overall and are not reliant on weighting or temporal aggregation. The ATT from an inverse hyperbolic sine count specification was statistically significant and indicated that managed care was associated with an 8.8% (95% CI, 2.1%-15.6%; *P* = .01) increase in ED visits. This increase is slightly lower than the 11.3% increase identified in the main specification because it does not account for population shifts and compares the effect to posttreatment, rather than pretreatment, rates. ATTs from the 3 models using alternative approaches to correct for staggered rollout biases (Wooldridge,^39^ Sun and Abraham,^45^ and Gardner^46^) were consistent in direction and magnitude. Taken together, these findings support the robustness of the main results.

**Table 3.** Comparison of Overall Difference-in-Differences Estimates of the Association Between Introducing Managed Care and Nontraumatic Dental Condition (NTDC) Emergency Department (ED) Visits and Charges Using Alternative Specifications^a^

Specification	Quarterly NTDC ED visits per 100 000 pediatric Medicaid enrollees		Mean charge for NTDC ED visits, $		Change in quarterly NTDC ED visits, %	
	Coefficient estimate (95% CI)	*P* value	Coefficient estimate (95% CI)	*P* value	Coefficient estimate (95% CI)	*P* value
Main (Callaway and Sant’Anna^41^)	14.951 (5.352 to 24.549)	.002	−49.09 (−143.15 to 44.97)	.31	NA	NA
Simplified 2 × 2	17.738 (5.664 to 29.812)	.006	13.06 (−102.96 to 129.08)	.82	NA	NA
IHS count	NA	NA	NA	NA	8.8 (2.1-15.6)	.01
Wooldridge^39^	19.113 (7.517 to 30.709)	.001	−27.92 (−106.72 to 50.88)	.49	NA	NA
Sun and Abraham^45^	15.156 (5.299 to 25.013)	.003	−48.32 (−140.98 to 44.34)	.30	NA	NA
Gardner^46^	15.526 (6.449 to 24.603)	<.001	−20.65 (−128.58 to 87.28)	.70	NA	NA

Abbreviations: IHS, inverse hyperbolic sine; NA, not applicable.

^a^ Data include 67 counties in Florida, 1206 county-quarters, and 34 414 NTDC ED visits. Difference-in-differences coefficient estimates are shown with corresponding 95% CIs. For the main and count IHS specifications, county-clustered multiplier bootstrap was used to calculate standard errors and corresponding 95% CIs, for the 2 × 2 specification no standard error adjustment was used, and for other specifications standard errors were clustered at the county level. For event study specifications, overall average treatment effects on the treated are constructed as a weighted average of all posttreatment groups.

## Discussion

Florida Medicaid has long struggled to deliver pediatric dental care, with service rates among the lowest in the nation. In 2010, 16% of Florida dentists participated in Medicaid,^47^ and only 24% of children enrolled in Florida Medicaid had any dental visit.^1^ A decade later, these figures were only slightly improved—22% of dentists participated^3^ and 35% of children had a dental visit that year.^1^ We examined the association between the introduction of fully capitated managed care for dental services and the rate of NTDC ED visits among Medicaid-enrolled children in Florida. Using a DiD specification we found that in the first 2.5 years after implementation, the introduction of managed care was associated with an 11.3% (95% CI, 4.0%-18.4%) increase in NTDC ED visits. The average charges for these visits did not change.

We observed an immediate increase in NTDC ED visits following the implementation of managed care, with visits continuing to rise over time. This abrupt increase is counterintuitive given that dental conditions can take years to progress. However, most children enrolled in Florida’s Medicaid program do not regularly receive preventive dental care.^1^ The immediate change in visits may reflect a substitution toward ED care: children who previously saw a dentist now present to the ED instead. Similar patterns have been observed in other settings. For example, when Medicaid adult emergency dental coverage was eliminated in Maryland, dental ED visits increased by 22%.^48^ Additionally, EDs are used as substitutes for medical services when access to primary care is limited.^49^ The longer-term increases in NTDC ED visits observed in the second year of implementation could be due to decreased preventive care increasing the overall burden of unaddressed dental disease in the population.

The present findings are consistent with prior research, which found that the adoption of managed care for Medicaid dental services was associated with fewer visits to dentists.^14^ There are several reasons why the introduction of managed care could decrease dental care access. One possibility is that some dentists who previously accepted FFS Medicaid may have chosen not to participate in a managed care network. This explanation seems unlikely, as Florida’s Workforce Survey of Dentists found no substantial reduction in the overall number of dentists accepting Medicaid immediately following the adoption of managed care.^50^

However, even if the overall number of dentists accepting Medicaid did not decline under managed care, patients likely experienced less flexibility in their choice of dentist. Under FFS, patients could choose any dentist who accepted Medicaid. In contrast, the managed care model used in Florida restricted patients to assigned in-network dentists, although they retained the option to switch between in-network dentists. Surveys of patients enrolled in Florida Medicaid have shown that confusion around which dentists are in-network is one of the biggest barriers to accessing care.^5^ Enrollees may have struggled to navigate the transition if they were assigned to a new dentist, especially if they lacked transportation options or struggled to trust a new dentist.

Although the data used in this study are relatively old, covering 2010 to 2014, the findings are relevant to ongoing policy decisions. There is no consensus on the best payment system for Medicaid’s dental benefits, with states approximately evenly split between FFS programs, managed care programs administered by medical plans (carve in), and managed care programs administered by dental plans (carve out).^51^ As of 2021, 10 states provided Medicaid dental benefits to the majority of enrollees through capitated payments to dental plans.^52^ As of the end of 2018, 18 states offered pediatric Medicaid dental services FFS and could transition to managed care in the future.^14^ Since then, several states have initiated or considered managed care programs for dental services. In 2018, Washington released a request for dental plans as part of implementing a Medicaid managed care program^15^ before canceling the program less than a year later.^53^ As of 2023 in North Carolina, a taskforce of dentists, Medicaid officials, and academics was investigating whether the state should implement dental managed care.^16^ In February 2024, Oklahoma transitioned its FFS dental program to managed care.^17,18^ In addition, Florida continues to change how it pays for Medicaid dental benefits. In 2018, the state carved out dental benefits for children and adults, separating them from medical coverage. Whether dental benefits should be managed by dental or medical plans remains contentious, with the AHCA presenting uncontrolled time series analysis in support of reintegrating dental benefits into medical plans (a carve in) in 2021,^54^ which was considered in the Florida legislature in 2022.^55,56^ State program details are provided in the eAppendix in Supplement 1.

### Limitations

This study has several limitations. First, NTDC ED visits are relatively uncommon, leading to a modest sample size for the analysis and wide confidence intervals for the ATTs. However, NTDC ED visits are frequently used in research because these visits are typically preventable, indicative of inadequate access to regular dental care, often result in only palliative treatment, and can lead to inappropriate opioid use.^31,57,58^ Second, the primary results relied on an assumption that the posttreatment trends in already-treated control counties are an accurate counterfactual for posttreatment trends in treatment counties. We support this assumption by showing that the trends in these groups were similar before policy implementation, verifying that no treatment dynamics remained from pilot implementations 5 years earlier. Third, the results for charges were based on what ED departments charged, not what was reimbursed by Medicaid. If the proportion of charges that are reimbursed changed alongside the introduction of managed care, then the conclusions about mean charges could be misleading and may not apply to mean reimbursements. Fourth, we relied on Medicaid population estimates from the ACS, rather than enrollment files. We explore the impact of systematic errors in population estimates in eTable 2 in Supplement 1.

Further research should focus on whether the adoption of managed care for dental services changed overall dental and medical costs and whether managed care delivered the promised cost savings to states. Finally, there is a need for research on whether managed care dental services are best administered by medical plans or dental plans.

## Conclusions

This cohort study highlights the potential unintended consequences of adopting managed care in Medicaid. Introducing managed care was associated with an increase in ED visits for preventable dental conditions, suggesting decreased access to dentists. Managed care policies are attractive because they theoretically enable states to control spending and transfer budgetary risk to the private sector.^59^ However, if states want to prioritize dental care access for children,^13^ then they may consider retaining FFS payment models.

## Notes

Supplement 1.
eAppendix

eReferences
**eTable 1.** Mean percentage difference between ACS 1-year and ACS 5-year estimates by year offset **eTable 2.** Comparison of population estimates **eTable 3.** Comparison of primary vs primary and secondary diagnosis codes **eTable 4.** Overall and quarterly event study estimates of the relationship between introducing managed care and NTDC ED visits and charges. **eFigure 1.** Relationship between ACS 1-year estimates, offset ACS-5 year estimates, and extrapolated data **eFigure 2.** Enrollment in Pre-paid Dental Health Plans by medical Medicaid coverage type and county

Supplement 2. Data Sharing Statement
