# Supplementary material for: Medicaid Managed Care and Pediatric Dental Emergency Department Visits
Source: JAMA Health Forum. 2024 Jun 14;5(6):e241472. doi: 10.1001/jamahealthforum.2024.1472 (PMC11179125; doi:10.1001/jamahealthforum.2024.1472)
Supplement: Supplement 1. — eAppendix eReferences eTable 1. Mean percentage difference between ACS 1-year and ACS 5-year estimates by year offset eTable 2. Comparison of population estimates eTable 3. Comparison of primary vs primary and secondary diagnosis codes eTable 4. Overall and quarterly event study estimates of the relationship between introducing managed care and NTDC ED visits and charges. eFigure 1. Relationship between ACS 1-year estimates, offset ACS-5 year estimates, and extrapolated data eFigure 2. Enrollment in Pre-paid Dental Health Plans by medical Medicaid coverage type and county [file jamahealthforum-e241472-s001.pdf]

Supplemental Online Content

Baker L, Munnich EL, Kranz AM. Medicaid managed care and pediatric dental emergency department visits. *JAMA Health Forum*. 2024;5(6):e241472. doi:10.1001/jamahealthforum.2024.1472

eAppendix

eReferences

eTable 1. Mean percentage difference between ACS 1-year and ACS 5-year estimates by year offset

eTable 2. Comparison of population estimates

eTable 3. Comparison of primary vs primary and secondary diagnosis codes

eTable 4. Overall and quarterly event study estimates of the relationship between introducing managed care and NTDC ED visits and charges.

eFigure 1. Relationship between ACS 1-year estimates, offset ACS-5 year estimates, and extrapolated data

eFigure 2. Enrollment in Pre-paid Dental Health Plans by medical Medicaid coverage type and county

This supplemental material has been provided by the authors to give readers additional information about their work.

eAppendix

Census Medicaid population

County-level populations were estimated using the American Community Survey 5-year data.[1] For each county, we estimated the number of under-18-year-old Medicaid enrollees as the sum of:

- B27010_007: under 18-year-olds with Medicaid only
- B27010_013: under 18-year-olds with Medicaid and Medicare only
- B27010_015: under 18-year-olds with another combination of public insurance.

The 5-year data were chosen over 1-year data because the larger sample size improves the accuracy of the population estimate and the 1-year data excludes geographies that have populations smaller than 65,000, including many Florida counties. However, because 5-year data is collected over an extended period (e.g. from 2009-2013), it is unclear if it is representative of a single year.

To identify what year is best represented by the 5-year ACS estimates, we compared them to the 1-year ACS estimates in counties with populations over 65,000. The 5-year estimates were available for collection periods ending 2013-2021 and 1-year estimates were available 2013-2019. We calculated the mean percentage difference in the 5-year and 1-year estimates of the number of Medicaid enrollees under 19 across lags from -5 years to +5 years for each Florida County. We then took the mean of these results across all counties to yield our estimate of the percentage difference, shown in eTable 1.

Using a lag of 2 years for the 5-year survey estimates minimized the error with 1-year estimates, which was close to zero (0.1%). For instance, the 2009-2013 survey was chosen to represent 2011. This makes intrinsic sense: 2011 is the midpoint of the survey period, so if trends are approximately linear, then it will be representative of that year.

Lagging the 5-year survey estimates by 2 years provided annual estimates for county populations from 2011 to 2019. Our ED outcome data was for 2010-2014 at a quarterly frequency. To interpolate quarterly values, we assigned the estimates for a year to the third quarter (so 2011 estimates were coded as 2011.5) and then linearly interpolated between observations. Medicaid population variables first became available in the 2009-2013 ACS survey. To extrapolate outside the range of the data, we fitted thin-plate splines with 9 degrees of freedom to each county's observations between 2011 and 2019. We then sampled the values of these splines for the 2010 data.

Figure A1 shows 1-year ACS estimates (blue), offset 5-year ACS estimates (orange), and the interpolated/extrapolated dataset (green). The interpolated data is obscured by the 5-year estimates because they correspond exactly. Counties with small populations toward the bottom of the plot have no 1-year data because this data is only available for counties with at least 65,000 population.

Population age sample

The ACS reports insured-population data for discrete age groups, in our case, for children aged 0 – 17 years. This does not exactly match with the population affected by Florida's managed care policy, which applied to all enrollees aged 0 – 20 years. To ensure that our rate numerator and denominator used the same population, we limited our emergency department visit sample

© 2024 Baker L et al. *JAMA Health Forum.*

to visits from Medicaid-enrolled children aged 0 – 17 years. Therefore, our results apply only to children aged 0 – 17 years, rather than all enrollees aged 0 – 20 years.

Robustness analyses

As robustness checks on our primary outcome results, we estimated several alternative model specifications. Specifically, we aggregated the county-quarter observations into a simplified 2x2 DiD model with two time periods (pre- and post-intervention) and two groups (treatment and control). In this specification, we excluded the transition quarters from 2012-Q1 to 2012-Q4 when managed care programs were implemented in some, but not all, treatment counties. This simplified approach does not require population weighting or aggregation of time-specific ATTs and serves as a straightforward check on our main results. No standard error clustering correction was used, since observations are already aggregated.

We also present results using NTDC ED visit counts, rather than NTDC ED visit rates, to verify that changes in rates are driven by changes in the number of visits (the numerator) rather than changes in the Medicaid population (the denominator). Since Florida counties vary greatly in size, we compare percentage changes in counts, rather than absolute changes in counts.[2] To do this, we use the inverse hyperbolic sine (IHS) transformation of NTDC ED visit counts as the outcome variable. As with log transformations, regression coefficients from IHS-transformed models can be interpreted as approximate percentage changes. However, unlike the log transformation, the IHS transformation is defined at zero.[3] As in the main specification, we used the two-way fixed effects (TWFE) approach recommended by Callaway and Sant'Anna[4] and multiplier bootstrapping for standard errors.

There are several methods to correct for staggered treatment bias in TWFE by excluding invalid group-time comparisons. We chose the method proposed by Callaway and Sant'Anna[4] because it imposes a less stringent parallel trends assumption than alternatives while still generating pre-treatment ATTs to evaluate parallel trends.[5] Because there is no consensus on the optimal approach for adjusting TWFE estimates in the presence of staggered rollouts,[5] we present ATTs from three alternative methods (Wooldridge, Gardner, Sun and Abraham) as a robustness check.[6–8] For these specifications, standard errors were clustered at the county level.

Population Variation

ACS population estimates contain uncertainty because they are based on a population sample. The ACS reports a margin of error (MoE) for each population value. To identify whether population uncertainty impacts our results, we performed our primary analysis with low and high population values based on these MoEs, which are equivalent to 90% confidence intervals. We estimated the MoE of sums as the root sum of squares of constituent MoEs.[9] Results from these analyses are shown in eTable 2.

With the low population estimates the rate denominator is smaller, so the overall rate effect is larger. The converse is true for high population estimates. However, the percentage change relative to the pre-treatment mean in treated counties is similar for all these analyses, because the denominator impacts the pre-treatment as well as post-treatment rates. We therefore believe that the interpretation of our results is relatively robust to systematic biases in population estimates.

© 2024 Baker L et al. *JAMA Health Forum.*

Principal Diagnoses

Our main specification used ED visits that have either a principal or secondary NTDC diagnosis. Secondary NTDC ED visits may be less strongly associated with the adoption of managed care than principal visits. To test this, we subsetted our data to only visits with a principal NTDC diagnosis, dropping 22.1% of our original sample which only has a secondary NTDC diagnosis code. We then reran our main specification for our primary outcome. The results are shown in eTable 3.

The absolute change in ED visit rates was smaller for the specification with only primary visits, but this is because it is a smaller sample of all visits. When these rate changes are compared as percentages of the pre-treatment mean in treated counties, the change is slightly larger when only considering principal visits. This makes intrinsic sense: if the adoption of managed care is impacting NTDC ED visits, then it ought to have a slightly larger effect on primary visits than on secondary visits. These results corroborate our main analysis, providing additional evidence that NTDC ED visits are associated with the adoption of managed care.

State Medicaid Policies

Nasseh and Bowblis[10], used Medicaid data from 2014-2018 to categorize states as fee-for-service (FFS) if the percentage of pediatric dental claims which were FFS was at or near 100%. As of 2018, they categorized 18 states as being fee-for-service:

> Alabama, Alaska, Connecticut, Delaware, Hawaii, Massachusetts, Maryland, Maine, Montana, North Carolina, North Dakota, New Hampshire, Oklahoma*, South Carolina, South Dakota, Tennessee, Virginia and Washington State.

*Oklahoma switched from FFS to a capitated dental managed care plan in February 2024[11,12]

MACPAC collects data on the percentage of enrollees in different forms of managed care programs by state. As of 2021: 14 states provided provide Medicaid dental benefits to some enrollees through capitated dental managed care plans, of which 10 provide dental benefits to the majority of their enrollees.[13] The states in which Medicaid dental services are provided through capitated dental managed care plans to some enrollees are:

> Arkansas†, California, Florida†, Idaho†, Iowa†, Louisiana†, Michigan, Nebraska†, Nevada†, Oregon, Rhode Island, Tennessee†, Texas†, Utah†.

†Denotes that the majority of enrollees are covered by capitated dental managed care plans.

© 2024 Baker L et al. *JAMA Health Forum.*

eReferences

1. U.S. Census Bureau. American Community Survey 5-Year Data (2009-2021). Published online 2022. Accessed August 9, 2023. https://www.census.gov/data/developers/data-sets/acs-5year.html

2. Finkelstein A. The Aggregate Effects of Health Insurance: Evidence from the Introduction of Medicare*. *Q J Econ*. 2007;122(1):1-37. doi:10.1162/qjec.122.1.1

3. Burbidge JB, Magee L, Robb AL. Alternative Transformations to Handle Extreme Values of the Dependent Variable. *J Am Stat Assoc*. 1988;83(401):123-127. doi:10.2307/2288929

4. Callaway B, Sant'Anna PHC. Difference-in-Differences with multiple time periods. *J Econom*. 2021;225(2):200-230. doi:10.1016/j.jeconom.2020.12.001

5. de Chaisemartin C, D'Haultfœuille X. Two-way fixed effects and differences-in-differences with heterogeneous treatment effects: a survey. *Econom J*. 2023;26(3):C1-C30. doi:10.1093/ectj/utac017

6. Wooldridge JM. Two-Way Fixed Effects, the Two-Way Mundlak Regression, and Difference-in-Differences Estimators. Published online August 17, 2021. doi:10.2139/ssrn.3906345

7. Sun L, Abraham S. Estimating dynamic treatment effects in event studies with heterogeneous treatment effects. *J Econom*. 2021;225(2):175-199. doi:10.1016/j.jeconom.2020.09.006

8. Gardner J. Two-stage differences in differences. Published online July 12, 2022. doi:10.48550/arXiv.2207.05943

9. American Community Survey. *Worked Examples for Approximating Standard Errors Using American Community Survey Data*.; 2022. https://www2.census.gov/programs-surveys/acs/tech_docs/accuracy/2022_ACS_Accuracy_Document_Worked_Examples.pdf

10. Nasseh K, Bowblis JR. The effect on dental care utilization from transitioning pediatric Medicaid beneficiaries to managed care. *Health Econ*. 2022;31(6):1103-1128. doi:10.1002/hec.4496

11. Oklahoma Health Care Authority. About SoonerSelect. Published December 15, 2023. Accessed January 4, 2024. https://oklahoma.gov/ohca/soonerselect/about.html

12. Oklahoma Health Care Authority. *SoonerSelect Dental Program Request for Proposals*.; 2022. https://oklahoma.gov/content/dam/ok/en/okhca/docs/about/procurement/soonerselect-dental-8070001412/documents/Dental%20RFP%20Amendment_3_10.27.22%20Final.pdf

13. MACPAC. *MACStats: Medicaid and CHIP Data Book*.; 2023. https://www.macpac.gov/publication/macstats-medicaid-and-chip-data-book-2/

© 2024 Baker L et al. *JAMA Health Forum.*

eTables

eTable 1. Mean percentage difference between ACS 1-year and ACS 5-year estimates by year offset

Offset for 5-year ACS	Mean percentage difference
-5	7.6%
-4	4.5%
-3	2.5%
**-2**	**-0.1%**
-1	-2.0%
0	-3.3%
1	-5.0%
2	-6.9%
3	-8.5%
4	-10.2%
5	-10.8%

Note: Bolded value indicates absolute minimum error. ACS: American Community Survey.

© 2024 Baker L et al. *JAMA Health Forum.*

eTable 2. Comparison of population estimates

Population	Quarterly NTDC ED visits per 100,000 pediatric Medicaid Enrollees	Pre-treatment mean in treatment counties	Percentage change
**ACS High**	14.172***<br>[5.1178, 23.226]	125.252	11.31%
**ACS point estimate**	14.951***<br>[5.352, 24.549]	132.677	11.27%
**ACS Low**	15.790***<br>[5.669, 25.9119]	141.038	11.20%

Note: Difference-in-differences coefficient estimates are shown with corresponding 95% confidence intervals in brackets. ATT: Average treatment effect on the treated; ED: Emergency department; NTDC: Non-traumatic dental condition. ***p < 0.01, **p < 0.05, *p < 0.1

© 2024 Baker L et al. *JAMA Health Forum.*

eTable 3. Comparison of primary vs primary and secondary diagnosis codes

Sample	Quarterly NTDC ED visits per 100,000 pediatric Medicaid Enrollees	Mean ED visit rate in treatment counties before treatment	Percentage change
Primary and secondary visits	14.951*** [5.352, 24.549]	132.67	11.27%
Primary visits only	12.849*** [3.531, 22.166]	103.89	12.37%

Note: Data include 67 counties, 1206 county-quarters, and 34,414 NTDC ED visits. Difference-in-differences coefficient estimates are shown and corresponding 95% confidence intervals are in brackets. Overall estimates are constructed as a weighted average of all post-treatment groups. ED: Emergency department; NTDC: Non-traumatic dental condition. ***p < 0.01, **p < 0.05, *p < 0.1

© 2024 Baker L et al. *JAMA Health Forum.*

eTable 4. Overall and quarterly event study estimates of the relationship between introducing managed care and NTDC ED visits and charges

Quarter	Quarterly NTDC ED visits per 100,000 pediatric Medicaid Enrollees	Mean Charge for NTDC ED visits ($)
**Overall**	14.951***<br>[5.3527, 24.549]	-49.087<br>[-143.15, 44.972]
**-2.75**	-2.818<br>[-19.368, 13.732]	0.16802<br>[-134.48, 134.82]
**-2.5**	-9.6953<br>[-28.003, 8.6125	-35.267<br>[-194.54, 124.01]
**-2.25**	-8.9239<br>[-28.662, 10.815]	-38.08<br>[-193.55, 117.39]
**-2**	1.028<br>[-13.196, 15.252]	-3.4569<br>[-162.4, 155.48]
**-1.75**	-7.0499<br>[-19.038, 4.9379]	-101.89<br>[-317.28, 113.51]
**-1.5**	2.8707<br>[-8.4186, 14.16]	-66.881<br>[-208.03, 74.268]
**-1.25**	-0.64422<br>[-15.185, 13.896]	-73.452<br>[-192.85, 45.947]
**-1**	0.084213<br>[-13.3, 13.469]	0.67945<br>[-124.9, 126.26]
**-0.75**	1.8102<br>[-7.2976, 10.918]	-52.364<br>[-156.67, 51.942]
**-0.5**	2.1501<br>[-12.074, 16.374]	2.3011<br>[-87.276, 91.878]
**-0.25**	0<br>[NA, NA]	0<br>[NA, NA]
**0**	11.844*<br>[-0.094183, 23.782]	-142.3**<br>[-260.86, -23.739]
**0.25**	15.765**<br>[1.8573, 29.673]	-64.726<br>[-203.36, 73.912]
**0.5**	10.444<br>[-4.7319, 25.62]	-141.06**<br>[-249.81, -32.307]
**0.75**	12.406**<br>[0.68098, 24.132]	-29.886<br>[-142.9, 83.132]
**1**	13.83*<br>[-0.10528, 27.765]	39.956<br>[-116.22, 196.13]
**1.25**	17.39**<br>[2.1745, 32.606]	50.996<br>[-95.066, 197.06]
**1.5**	26.174**<br>[6.0034, 46.344]	-117.16<br>[-311.48, 77.157]

© 2024 Baker L et al. *JAMA Health Forum.*

**1.75**	13.402<br>[-8.0646, 34.869]	137.93<br>[-397.53, 673.4]
**2**	15.69*<br>[-0.98638, 32.366]	22.435<br>[-244.18, 289.05]
**2.25**	24.261**<br>[0.56779, 47.955]	-63.99<br>[-240.93, 112.96]

Note: Data include 67 counties, 1206 county-quarters, and 34,414 NTDC ED visits. Difference-in-differences coefficient estimates are shown and corresponding 95% confidence intervals are in brackets. Overall estimates are constructed as a weighted average of all post-treatment groups. Pre- and post-treatment ATTs are constructed using long differences. ATT: Average treatment effect on the treated; ED: Emergency department; NTDC: Non-traumatic dental condition. ***p < 0.01, **p < 0.05, *p < 0.1

© 2024 Baker L et al. *JAMA Health Forum.*

eFigures

eFigure 1. Relationship between ACS 1-year estimates, offset ACS-5 year estimates, and extrapolated data

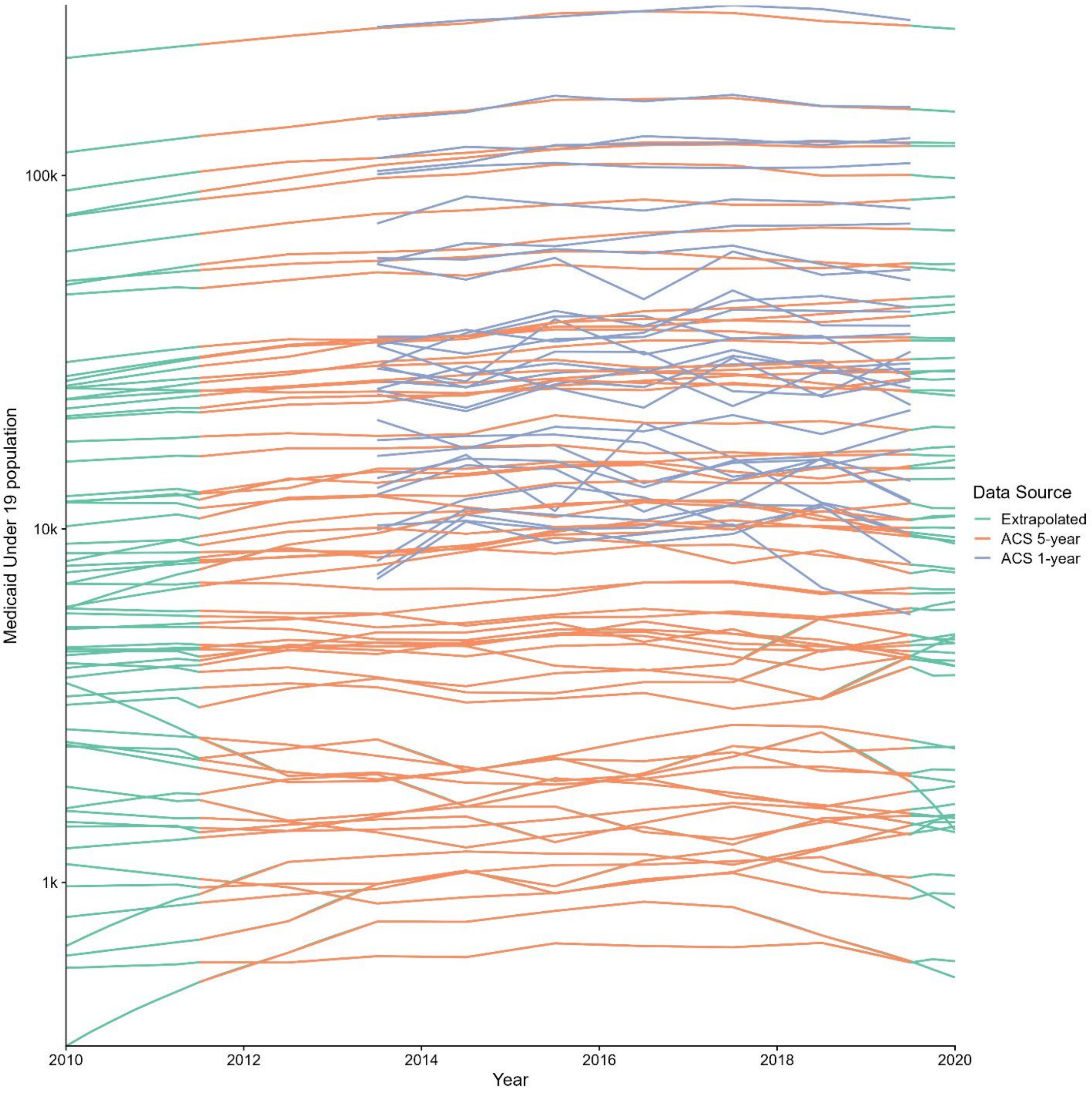

Note: Y-axis is shown on a log scale. All estimates have been offset by 0.5 years (so the 2011 survey is shown in 2011.5), to allow for quarter interpolation. ACS 5-year estimates have additionally been offset by 2 years based on the results in table A1. ACS: American Community Survey.

© 2024 Baker L et al. *JAMA Health Forum.*

eFigure 2. Enrollment in Pre-paid Dental Health Plans by medical Medicaid coverage type and county

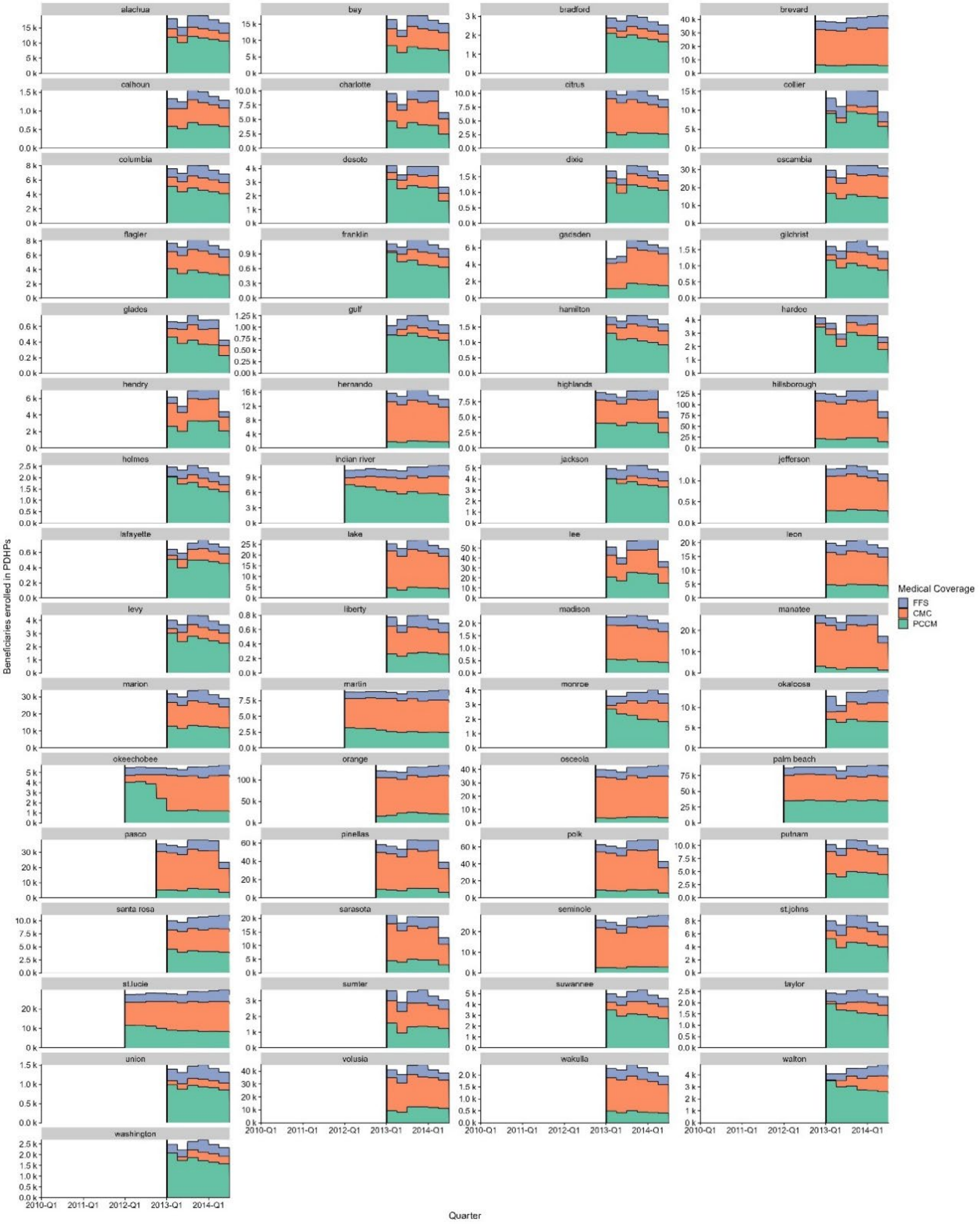

Note: Pre-paid Dental Health Plans were mandatory for all Medicaid enrollees under 21 years of age, irrespective of the medical coverage, so enrollees of all medical plan types were transitioned. CMC: Comprehensive managed care; FFS: Fee for service; PCCM: primary care case management

© 2024 Baker L et al. *JAMA Health Forum.*
