# Supplementary material for: Medicaid Managed Care and Pediatric Dental Emergency Department Visits
Source: JAMA Health Forum. 2024 Jun 14;5(6):e241472. doi: 10.1001/jamahealthforum.2024.1472 (PMC11179125; doi:10.1001/jamahealthforum.2024.1472)
Supplement: Supplement 2. — Data Sharing Statement [file jamahealthforum-e241472-s002.pdf]

Data Sharing Statement

Baker. Medicaid Managed Care and Pediatric Dental Emergency Department Visits. *JAMA Health Forum*. Published June 14, 2024. doi:10.1001/jamahealthforum.2024.1472

Data
Florida Emergency Department Visits
Data available: No
Explanation for why data not available: Data are owned by the Florida Agency for Health Care Administration. Data can be acquired upon application and payment to the Florida Agency for Health Care Administration.
American Community Survey 5-Year Data
Data available: No
Explanation for why data not available: These data are publicly available and can be obtained from the Census website or APIs.
Florida Medicaid Enrollment Reports
Data available: No
Explanation for why data not available: These data are publicly available and can be obtained from the Florida Agency for Health Care Administration website.
